# Neutralization of ionic interactions by dextran-based single-chain nanoparticles improves tobramycin diffusion into a mature biofilm

**DOI:** 10.1038/s41522-022-00317-9

**Published:** 2022-07-04

**Authors:** Núria Blanco-Cabra, Julie Movellan, Marco Marradi, Raquel Gracia, Cristian Salvador, Damien Dupin, Iraida Loinaz, Eduard Torrents

**Affiliations:** 1 Bacterial Infections: Antimicrobial Therapies group, Institute for Bioengineering of Catalonia (IBEC), The Barcelona Institute of Science and Technology (BIST), Barcelona, Spain; 2 Microbiology Section, Department of Genetics, Microbiology, and Statistics, Biology Faculty, Universitat de Barcelona, Barcelona, Spain; 3 CIDETEC, Basque Research and Technology Alliance (BRTA), Parque Científico y Tecnológico de Gipuzkoa, Donostia-San Sebastián, Spain; 4 Department of Chemistry “Ugo Schiff”, University of Florence, Sesto Fiorentino, FI Italy

**Keywords:** Biofilms, Applied microbiology

## Abstract

The extracellular matrix protects biofilm cells by reducing diffusion of antimicrobials. Tobramycin is an antibiotic used extensively to treat *P. aeruginosa* biofilms, but it is sequestered in the biofilm periphery by the extracellular negative charge matrix and loses its efficacy significantly. Dispersal of the biofilm extracellular matrix with enzymes such as DNase I is another promising therapy that enhances antibiotic diffusion into the biofilm. Here, we combine the charge neutralization of tobramycin provided by dextran-based single-chain polymer nanoparticles (SCPNs) together with DNase I to break the biofilm matrix. Our study demonstrates that the SCPNs improve the activity of tobramycin and DNase I by neutralizing the ionic interactions that keep this antibiotic in the biofilm periphery. Moreover, the detailed effects and interactions of nanoformulations with extracellular matrix components were revealed through time-lapse imaging of the *P. aeruginosa* biofilms by laser scanning confocal microscopy with specific labeling of the different biofilm components.

## Introduction

Antibiotics are the principal therapy against acute bacterial infections. However, when a bacterial community attaches to a surface and grows as a biofilm, the resulting infections become chronic, and antibiotics are much less effective^1^. Bacteria form biofilms by building an extracellular matrix, which creates a physical and chemical barrier that is the leading cause of antibiotic inefficiency. This extracellular biofilm matrix comprises polysaccharides, lipids, proteins, and extracellular DNA (eDNA)^2^.

*Pseudomonas aeruginosa* is a bacterium that usually forms biofilms in lungs affected by cystic fibrosis (CF) or chronic obstructive pulmonary disease (COPD). Apart from being the primary cause of morbidity and mortality for these illnesses^3^, *P. aeruginosa* is usually involved in chronic wound infections^4^ and is the leading cause of microbial keratitis^5^. *Pseudomonas* biofilms are a public health concern^6^ as the leading cause of nosocomial infections^7^, such as ventilator-associated pneumonia^8^ and catheter-associated urinary tract infections^9^. Moreover, *P. aeruginosa* is frequently associated with other bacteria, and these coinfections worsen the outcome of the disease^6,10^. eDNA is a key part of the *P. aeruginosa* extracellular biofilm matrix, as it promotes bacterial adhesion on the surface in the early stages, maintains its structural stability in mature biofilms^11–13^ and increases the viscosity of sputum in CF patients^14^. eDNA was thought to be only a residual material of bacterial lysis until it was reported that the enzyme deoxyribonuclease (DNase I) inhibits biofilm formation and dissolves established biofilms^15^. Currently, this enzyme (commercially available by Genentech with the name of Pulmozyme) is used to treat CF patients via aerosol administration, and it aids mucus clearance by reducing the viscosity and improving antibiotic chemotherapy^16^.

When a pulmonary infection of *P. aeruginosa* becomes chronic and forms a biofilm, it frequently converts to a mucoid phenotype by overproducing the anionic polysaccharide alginate in the extracellular matrix^17^. Alginate protects bacteria from the immune system by inhibiting the complement system and reducing phagocytosis by neutrophils and macrophages. Together with eDNA, this negatively charged polysaccharide binds to a positively charged antibiotic, thus diminishing antibiotic diffusion throughout the biofilm^18–20^.

Drug nanocarriers can be used to effectively overcome these barriers and transport the drug to the site of interest. Nanoparticles protect the antimicrobial agent from degradation or deactivation, prevent interactions between the drug and biofilm extracellular polymeric substances (EPS), and release the antibiotic locally and in a controlled manner to enhance antimicrobial activity.

Rational design of nanocarriers for pulmonary administration is key to reaching the biofilm resulting from a lung infection and delivering the cargo to the site of infection. Previous studies have demonstrated that to cross the mucus layer surrounding the infection, the size and surface charge of the delivery system are critical. Nanoparticles smaller than 500 nm in diameter and neutral or negatively charged are considered the best candidates^21–24^. However, for treatment of *P. aeruginosa* biofilms, it has been shown that the diffusion rate is size-dependent, and a nanosystem size below 100 nm is crucial^25^. Moreover, the mobility and diffusion rate of negatively charged nanoparticles are higher than those of positively charged nanoparticles, since the latter are retained by the anionic eDNA in the EPS^26^ via electrostatic interactions.

The most studied polymeric nanoparticles are based on chitosan and poly(lactic-*co*-glycolic acid) (PLGA), among others. The biocompatibility, degradability, and antimicrobial nature of chitosan make it a promising vector, but its hydrophobicity, which promotes aggregation under biological conditions, hinders its use. On the other hand, PLGA nanoparticles have the disadvantage of generating acidic degradation products and exhibiting low encapsulation efficiencies. Nevertheless, it is worth mentioning that PLGA loaded with ciprofloxacin/DNase I and colistin considerably reduced biofilm formation^27,28^. However, in both cases, the relatively small amount of antibiotic encapsulated, i.e., not higher than 1 wt%, indicates the need for administration of a very high concentration of the nanoformulation dose, which might jeopardize nebulization and rule out clinical use as a therapy for chronically infected patients.

Recently, single-chain polymer nanoparticles (SCPNs) based on dextran natural polysaccharides have been used to carry antimicrobial peptides to fight infections from antimicrobial-resistant bacteria^29^. SCPNs^30–35^ are obtained by collapsing a single polymer chain to form small nanoparticles with sizes generally reported to be below 20 nm, which are comparable to those of proteins and viruses. Functionalization can be readily modulated during or after production. Dextran-based SCPNs have shown ideal characteristics for use as nanocarriers in imaging and immunotherapy, as they are size-controlled, biocompatible, biodegradable, and water-dispersible^36,37^. Due to their small hydrodynamic sizes, homogenous lung distribution was observed by SPECT-CT after intratracheal administration using a Penn Century Microsprayer^®^ aerosolizer, indicating that these SCPNs are suitable nanocarriers for lung administration^29,37–39^.

In this work, SCPNs were used to carry tobramycin via electrostatic interactions, which neutralized the positive charges of the antibiotic, and they were formulated with DNase I to disaggregate mature *P. aeruginosa* biofilms. First, the maximum loading of the antibiotic and characterization of the nanoformulated nanoantibiotic were investigated to reach overall charge neutrality at the maximum nanoantibiotic loading capacity. The antimicrobial activities of the final candidates were studied in vitro in the presence of *P. aeruginosa*, and further, diffusion of the nanoantibiotics into the biofilm was investigated. Finally, the interactions of SCPNs, with or without tobramycin and/or DNase I, with a mature *Pseudomonas* biofilm matrix grown under continuous conditions mimicking those of chronic infections were studied through time-lapse imaging by laser scanning confocal microscopy.

## Results and discussion

### SCPN characterization and activity against planktonic bacteria

**Fig. 1:**
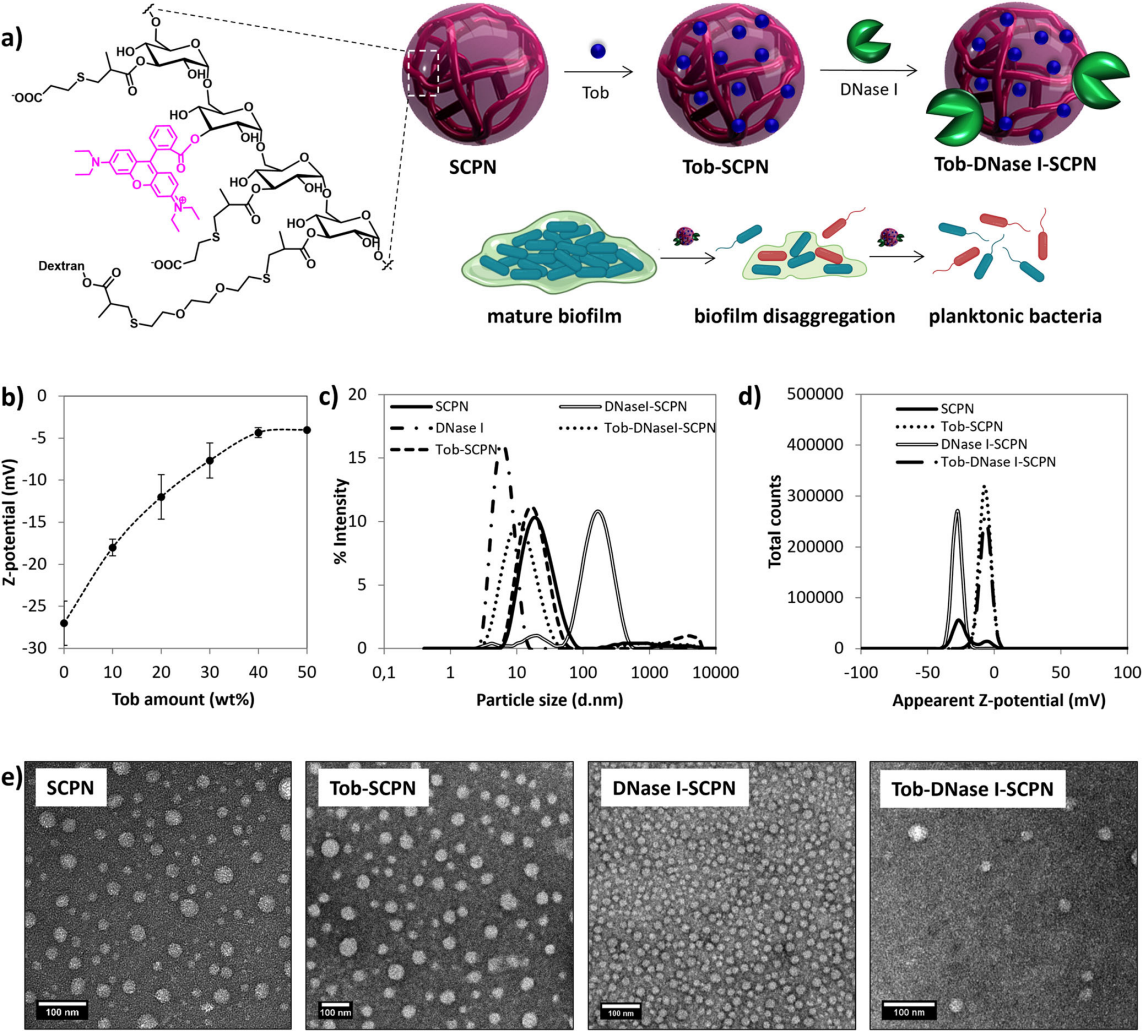
SCPN synthesis and characterization. **a** Schematic representation for the synthesis of negatively charged SCPN labeled with rhodamine (SCPN), formulation with tobramycin (Tob) and DNase I, and expected action on mature biofilm; (**b**) variations in zeta potential for Tob-SCPN formulations as a function of the amount of tobramycin (wt%) added, as judged by aqueous electrophoresis at 25 °C in the presence of 1 mM of NaCl. Error bars indicate the standard deviation between two experiments with at least three replicates of each experiment; (**c**) size distributions obtained by DLS at 10 mg/ml in PBS; (**d**) surface charges at 10 mg/ml and 1 mM NaCl for SCPN, DNase I, Tob-SCPN, DNase I-SCPN, and Tob-DNase I-SCPN dispersions; and (**e**) transmission electron micrographs of the 4 formulations dried at room temperature (scale bar represents 100 nm).

Dextran-based SCPNs (intermediate I, Supplementary Fig. 1) were prepared by controlled collapse of a single branched polymer coil into nanoparticles through intrachain cross-linking, as previously described^37^. Intermediate I was then labeled with rhodamine (Rh) via an esterification reaction. The progress of the reaction was followed by TLC, and the unreacted rhodamine was removed using a PD-10 Sephadex column. ^1^H NMR analyses of rhodamine-labeled SCPN, Intermediate II, showed the absence of ester hydrolysis and confirmed the presence of the Rh dye (Supplementary Fig. 2); UV–Vis spectroscopy was used to establish a concentration of 18 µg Rh/mg (Supplementary Fig. 3). Finally, the remaining methacrylate groups were functionalized with mercaptopropionic acid *via* thio-Michael addition to provide SCPNs containing anionic carboxylates at neutral pH. The hydrodynamic diameter of the functionalized SCPN was 19 (±1) nm, as judged by DLS (dynamic light scattering) (Table 1 and Supplementary Fig. 1). It is worth mentioning that a relatively high polydispersity index was obtained (0.26) due to polydispersity of the commercially available dextran polymer chains (see Supplementary Information). A nanoantibiotic SCPN with tobramycin (Tob-SCPN) was prepared by mixing an aqueous dispersion of SCPN with an aqueous solution of tobramycin, both at pH 7 (Fig. 1a). At this pH, the positively charged tobramycin (pK_a_s of the tobramycin amino groups = 7.4, 7.4, 6.2, 8.6, 7.6)^40^ was electrostatically attached to the negatively charged SCPN (pK_a_ = 4.9, determined experimentally). The electrostatic interactions between SCPN and tobramycin were monitored by aqueous electrophoresis, and a maximum 40 wt% loading of the antibiotic in Tob-SCPN was achieved until the cationic charges of tobramycin were neutralized by the nanoantibiotic. Increased zeta potential were observed from −21 (±1) mV obtained for SCPN, up to a quasi-neutral charge for Tob-SCPN (−5 ± 2 mV for Tob-SCPN, Table 1 and Fig. 1b), and this confirmed that both charged entities were neutralized via electrostatic interactions without affecting the size of the nanocarrier (Dh = 17 ± 1 nm) (Table 1 and Fig. 1d). Interestingly, it is anticipated that ~15 wt% of the nanoantibiotic can be nebulized due to the high loading of tobramycin in Tob-SCPN and its relatively low viscosity at such concentrations (close to water), and this corresponds to the concentration of Tob currently inhaled in clinical practice^41^. Finally, DNase I was directly formulated with SCPN and Tob-SCPN at 530 µg/mg and 410 µg/mg, respectively, to avoid any chemical modifications of the enzyme that could affect its activity (Fig. 1a). Aggregates with a Z-average of 102 nm were observed for DNase I-SCPN nanosystems, as judged by DLS (Fig. 1c and Table 1). In fact, the corresponding particle size distribution showed the presence of three different size populations, which might indicate the presence of free DNase I (6 nm particles) as well as SCPN with or without DNase I as single particles (15 nm particles) and some aggregates (100 nm particles). Actually, the slight decrease of the middle population for DNase I-SCPN at 15 nm compared to that for pristine particles (19 nm) could indicate the presence of enzyme bound to the particles via hydrogen bonding. However, it remains possible that all DNase I is free in solution. In addition, it is important to mention that DLS, an intensity-average technique, overestimates particle sizes in the presence of aggregates, as they scatter much more light. Thus, it is expected that the number of those aggregates was considerably lower than the relative distribution observed by DLS. In addition, it is noteworthy that a polydispersity index of 0.52 indicated that DLS was not the most suitable technique, and electron microscopy is indicated in this case, although the number of particles analyzed is much lower (over 100 particles). As shown in Fig. 1e, a transmission electron microscopy (TEM) image of DNase I-SCPN confirmed that the main particle size population was that for small particles with a number-average diameter (D_n_) of 21 (±5) nm. This number-average diameter is larger than the hydrodynamic diameter, which can be explained by flattening of the soft SCPN upon drying. Similar flattening was also observed by TEM after drying of SCPN, Tob-SCPN and Tob-DNase I-SCPN, which exhibited Dn values of 21 (±8), 37 (±13), and 37 (±13) nm, respectively.

**Table 1:** Compositions of the different formulations (SCPN: nude nanoparticles; Tob-SCPN: nanoparticles with 40 wt% tobramycin; DNase I-SCPN: SCPN formulated with DNase I; Tob-DNase I-SCPN: SCPN that contain 23 wt% tobramycin and DNase I).

SCPN type	Tobramycin content^a^ (µg Tob/mg SCPN)^b^	DNase I content^a^ (µg DNase I/mg SCPN)^b^	Number-average Diameter^c^ (Dn, nm)	Hydrodynamic Diameter (D_h_) / (d.nm)-PdI^d^	Z-potential (mV)^d^
SCPN	–	–	21 ± 8	19 ± 1/ 0.26 ± 0.04	−21 ± 1
Tob-SCPN	Total: 400 µg/mg Active: 400 µg/mg (100%)	–	37 ± 13	17 ± 1/ 0.27 ± 0.01	−5 ± 2
DNase I-SCPN	–	Total: 530 µg/mg Active: 30 µg/mg (5.6%)	21 ± 5	102 ± 2/ 0.52 ± 0.01	−28 ± 1
Tob-DNase I-SCPN	Total: 230 µg/mg Active: 230 µg/mg (100%)	Total: 410 µg/mg Active: 30 µg/mg (7.3%)	37 ± 13	11 ± 1/ 0.27 ± 0.07	−6 ± 1

^a^Tobramycin concentration measured by LC–MS.

^b^Tobramycin and DNase I activity measured by comparison of the free drug and soluble enzyme.

^c^TEM measurements (at least 100 particles counted) (Fig. 1e).

^d^Determined by aqueous electrophoresis (Fig. 1c, d).

On the other hand, a smaller particle size for Tob-DNase I-SCPN at 11 (±1) nm was observed by DLS, which could indicate interactions between the nanoparticles and the enzyme. The exact interaction mechanism remains unclear, and in addition to electrostatic interactions between the negative DNase I and some cationic tobramycin available at the surface of the particles (despite the overall negative charge of Tob-SCPN), other types of noncovalent interactions, such as H-bonding between SCPN, tobramycin and DNase I, could also occur (Fig. 1d).

**Fig. 2:**
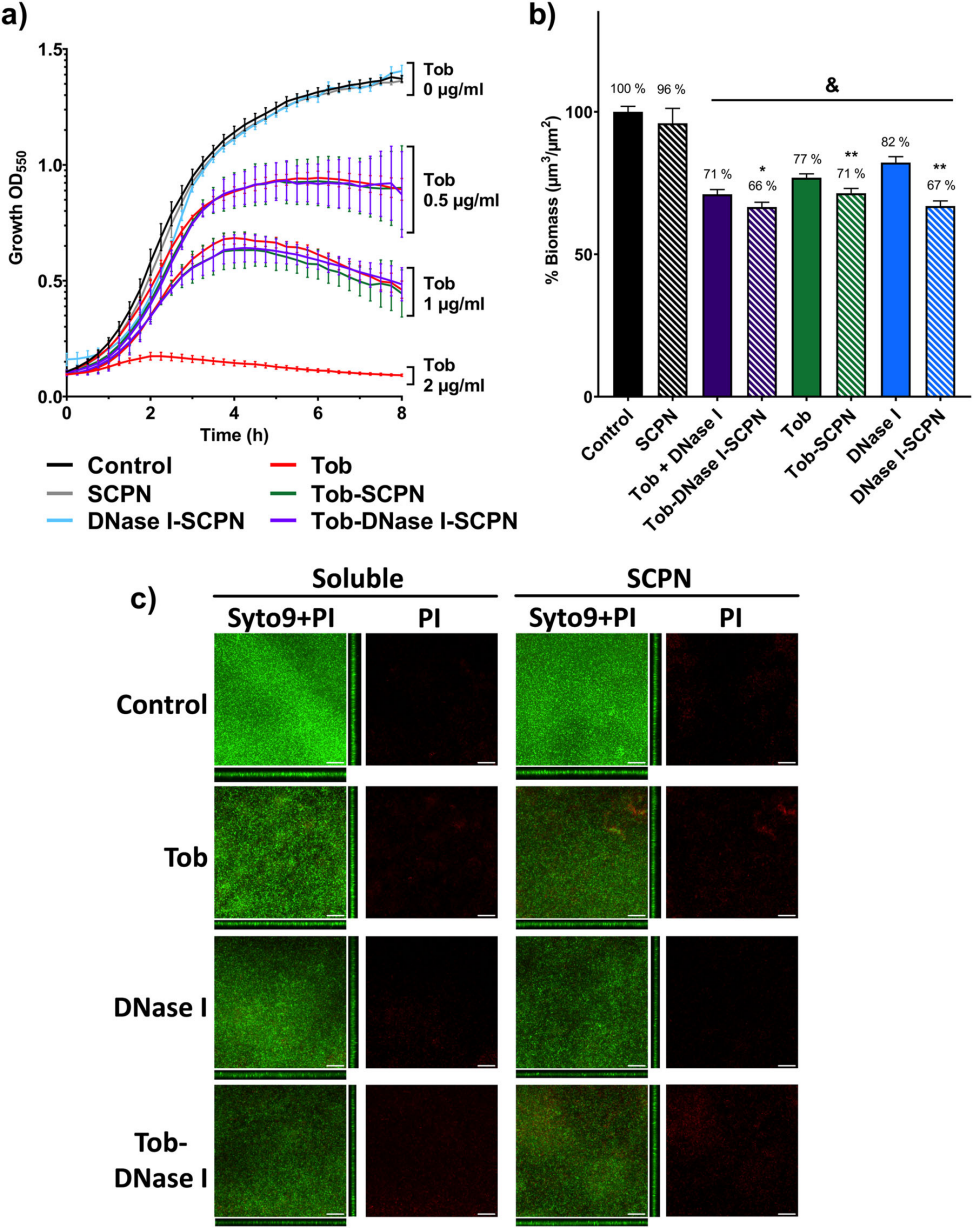
*P. aeruginosa* planktonic and biofilm treatments with different SCPNs. **a** Eight-hour bacterial growth curves for *P. aeruginosa* treated with soluble tobramycin (Tob, red) and different nanoparticles containing 0 µg/ml Tob (gray, 50 µg/ml SCPN; blue, 50 µg/ml DNase I-SCPN), 0.5 µg/ml Tob (green, 1.25 µg/ml Tob-SCPN; purple, 2.2 µg/ml Tob-DNase I-SCPN) and 1 µg/ml Tob (green, 2.5 µg/ml Tob-SCPN; purple, 4.3 µg/ml Tob-DNase I-SCPN). Black lines (control) represent growth without any treatment. Error bars indicate the standard deviation between three experiments with three replicates of each experiment. **b** Degradation of 72 h-old biofilms of *P. aeruginosa* treated for 16 h with 0.26 µg/ml DNase I and/or 2 µg/ml tobramycin (solid bars) and with nanoformulation containing SCPN with the corresponding concentration of both actives (striped bars). Blue, green and purple bars correspond to DNase I treatment, antibiotic tobramycin, and both treatments together, respectively. Data for the control (nontreated biofilm) and SCPN (without tobramycin and DNase I) are shown in black bars. All values are normalized against the control sample. Error bars indicate the standard deviation between two experiments with at least three replicates of each experiment. Statistical analyses were performed with the Student Unpaired *t* test (^&^*p* < 0.005 vs. Control, *p < 0.05; **p < 0.005 vs. treatment without SCPN) to determine significance. **c** Confocal laser scanning microscopy images of Live/Dead-stained *P. aeruginosa* biofilms. In each condition, it is shown the sum of the stack and the corresponding orthogonal views of merged live (Syto9) and dead (PI) staining and only the dead (PI) staining. The scale bar represents 40 µm.

The active concentrations of tobramycin and DNase I in the nanoformulations were further validated in two different experiments. First, the tobramycin activity was measured by comparing it with the same soluble free tobramycin concentration in an 8 h bacterial growth curve. As seen in Fig. 2a, the growth curves for *P. aeruginosa* planktonic treated with SCPN containing tobramycin showed the same growth pattern as those for treatment with free soluble tobramycin at equivalent concentrations. These results confirmed our capacity to quantify the tobramycin concentration accurately and showed that the antibiotic formulated with SCPN did not lose activity, even in the presence of DNase I. Moreover, the innocuousness of SCPN and DNase I-SCPN against planktonic bacteria was also confirmed, as the growth curve for treatment with these nanoparticles at high concentrations (50 µg/ml, Fig. 2a, gray and blue curves) did not differ from that seen without any treatment (Fig. 2a, black curves).

On the other hand, the activity of DNase I within the nanoformulation was verified by quantifying the DNA degradation activity in an agarose gel by electrophoresis and comparing it with the activity of soluble free DNase I (see Methods). In contrast to the activity of the 100% tobramycin SCPN cargo, the DNase I activity was only 5.6% and 7.3% for the total loaded DNase I in the DNase I-SCPN and Tob-DNase I-SCPN, respectively. Actually, it has been reported that enzyme inactivation by nanomaterials can also originate from large changes in protein structure following absorption or covalent binding, as well as from relatively low stability^42,43^.

### Formulation of tobramycin and DNase I with SCPN enhances the activity against continuous *P. aeruginosa* biofilms

A continuous-flow mature biofilm was used to measure antibiofilm activity, as it is one of the methodologies used to mimic the biofilms formed in chronically infected patients^44,45^. In this case, continuous biofilms of *P. aeruginosa* PAO1 were grown for 72 h in flow-cell chambers (see the Methods section) and then treated for 16 h with 0.26 µg/ml free DNase I, 2 µg/ml free tobramycin and a combination of both treatments. Note that the tobramycin MIC value for planktonic cells is 2 µg/ml (Fig. 2a). At the same time, the biofilms were treated with SCPN containing equivalent tobramycin and DNase I concentrations (5 µg Tob-SCPN/mL contained 2 µg/ml tobramycin, and 8.7 µg Tob-DNase I-SCPN/mL contained 2 µg/ml tobramycin and 0.26 µg/ml DNase I). The mature treated biofilms were stained and imaged under a confocal microscope, and different biofilm parameters were calculated (biomass and thickness).

As seen in Fig. 2b, the SCPN carrier had not effect on biofilm biomass (black striped bars), and the most effective treatment for biofilms was observed when Tob-DNase I-SCPNs containing both DNase I and tobramycin were used (purple striped bars); they reduced the volume of the formed biofilm by 34% (biomass) compared to the untreated biofilm (Fig. 2b, purple striped bars vs. black solid bars). Clearly, the nanoformulation with DNase I and/or tobramycin increased the activity of both molecules against the formed biofilm, as it improved the corresponding treatment compared to those of nonencapsulated compounds and showed 5 to 15% reductions of the established biofilm (from 71 to 66% with Tob-DNase I-SCPN, purple bars; from 77 to 71% with Tob-SCPN, green bars; and from 82 to 67% with DNase I-SCPN, blue bars).

It has been extensively reported that the use of enzymes for in vivo biofilm treatments without antibiotics leads to biofilm expansion and sepsis^46–48^. For the continuous biofilm shown in Fig. 2b, the constant media flow eliminated detached bacteria, which prevented biofilm regrowth. Therefore, even though reduction of the biofilm carried out by the SCPN containing DNase I (blue stripped bars) was almost the same as that of the SCPN containing both tobramycin and DNase I (purple stripped bars), it is crucial to add an antibiotic to increase treatment efficacy.

In addition to the reduction percentage for biomass in *Pseudomonas* biofilms treated with Tob-SCPN and Tob-DNase I-SCPN, notable increases in dead bacteria content (stained in red) were seen in the confocal laser scanning microscope (CLSM) images with a reddish nuance (Fig. 2c and Supplementary Table 1), which demonstrated the capacity of tobramycin to kill bacteria.

More importantly, it is worth emphasizing that, in addition to being continuous, biofilm formation over 72 h allowed the bacteria to grow in different layers, which caused the whole study to mimic in vivo conditions and made biofilm treatment more challenging. Most of the promising results published with nanoantibiotics were obtained with static biofilms^49–55^ or continuous biofilms grown for <24 h^56^. The biofilms resulting in these previous studies implied fewer bacteria in the biofilm state, often in monolayer, and corresponded to a weaker biofilm. Thus, less treatment was required to achieve full eradication compared to the mature biofilms found in infected patients, which indicated a structured and complex extracellular biofilm matrix. Additionally, it is noteworthy that the current study was performed with a single dose treatment, and further continuous treatment within our experimental setup would be required to considerably diminish the strong biofilm developed.

### SCPN formulated with DNase I enhances tobramycin penetrability into the *P. aeruginosa* biofilm matrix

**Fig. 3:**
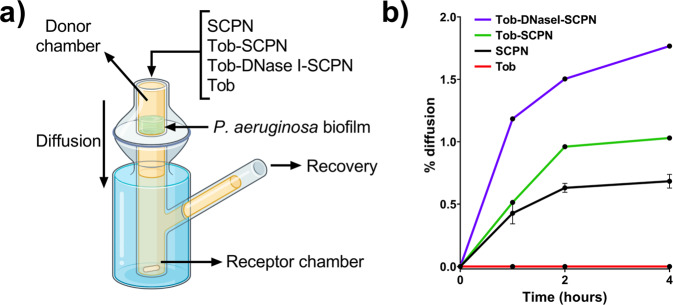
Permeability assay of SCPN diffusion into the *P. aeruginosa* biofilm. **a** Scheme of the Franz cell. **b** % diffusion of different SCPNs: SCPN (without tobramycin and DNase I) (black line), Tob-SCPN (green line), Tob-DNase I-SCPN (purple line), and free tobramycin (Tob, red line). Error bars indicate the standard deviation between three replicates of two different experiments.

Permeability studies of the different SCPNs in a *P. aeruginosa* mature biofilm were performed using a Franz diffusion cell (see scheme in Fig. 3a). This methodology allowed us to analyze nanoparticle and antibiotic permeability into a 72 h-old *P. aeruginosa* biofilm by quantifying the amounts of SCPNs and tobramycin that had diffused throughout the biofilm after 4 h. Soluble tobramycin recovery was 0% in all tested samples (Fig. 3b, red line). On the other hand, it was found that some of the labeled SCPNs were recovered after 4 h in all the nanoformulations tested. SCPN recovery efficiencies were 0.6, 1 and 2% for SCPN, Tob-SCPN and Tob-DNase I-SCPN, respectively (Fig. 3b, black, green and purple lines, respectively). The recovery of the SCPNs alone without the tobramycin indicates that the antibiotic was released from the SCPNs.

This experiment demonstrated that SCPNs diffused through the biofilm at a very low diffusion rate, while tobramycin was retained in the biofilm extracellular matrix. Nonetheless, considering the improvement in biofilm eradication that SCPNs conferred on tobramycin (Fig. 2b), it is suggested that SCPNs improved tobramycin entrance into deeper locations in the biofilm, which allowed more efficient killing of the embedded bacterial cells. This is in good agreement with the results of Thorn et al.^57^ who observed tobramycin immobilized in the upper portion of the biofilm over time. Moreover, they also showed that their nanoparticle formulation did not diffuse through the full thickness of the biofilm but enhanced tobramycin penetration.

### SCPN interacts with the biofilm extracellular matrix

The previous experiments (Figs. 2, 3) determined SCPN efficacy by analyzing biofilm biomass changes after 16 h of treatment and penetrability of the SCPNs and tobramycin into the biofilm, but none of them described the dynamics for modifications at the biofilm extracellular matrix level. Additionally, these studies did not show the progression of the treatment or the interactions of the nanoparticles with the biofilm. To further investigate this behavior, different SCPN interactions with an established *P. aeruginosa* PAO1 72 h-old continuous biofilm were monitored by imaging with time-lapse confocal microscopy. During the 16 h experiment, bacterial cells, eDNA, and the polysaccharides present in the biofilm extracellular matrix, were differentially stained and tracked. Three different nanoparticles (SCPN, Tob-SCPN, and Tob-Dnase I-SCPN) were labeled with rhodamine dye and inoculated inside the biofilm flow-cell chamber, all with the same nanoformulation concentration (100 µg of nanoformulation/ml). At this concentration, which was ten times higher than the one used in Fig. 2b, some aggregates with sizes of ~1–2 µm were observed after direct interaction with the biofilm structure. It is also noteworthy that most of the aggregates observed after contact with the biofilm tended to disappear over time, which indicated redispersion of the nanoparticles in the matrix. However, due to the limit of detection of the confocal microscopy technique, which can only detect particles with diameters >200 nm, the presence of those aggregates allowed tracking of the nanoantibiotic via confocal microscope imaging and enabled monitoring of their behavior over the 16 h of the time-lapse experiment. In this case, tracking these large nanoantibiotic aggregates represented the worst-case scenario; better penetration of nonaggregated nanoantibiotics into the bacterial biofilm could be anticipated.

Labeled SCPN-based nanoformulations and different dyes for staining bacterial cells, eDNA, and the extracellular matrix polysaccharides were simultaneously injected into a mature biofilm (see Methods). Then, different regions with ~1–2 µm SCPN aggregates were actively selected for tracking and imaging with the confocal microscope. The areas of interest were imaged for 16 h with the programmable acquisition algorithm of the Zen software with multiposition time series (Zeiss). Focus was maintained during imaging, and artifacts caused by thermal drift or vibrations were avoided by using Definite Focus equipment (Zeiss) coupled to the confocal microscope.

**Fig. 4:**
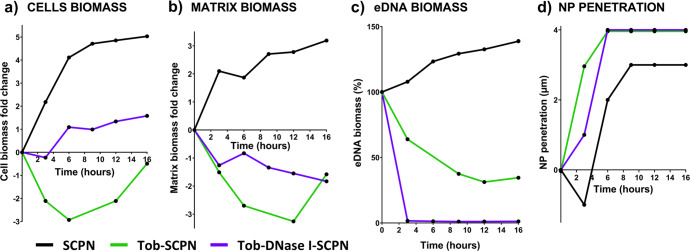
Biofilm behavior components with time. Representative experiments on biomass variation over time in (**a**) cells, (**b**) matrix, and (**c**) extracellular DNA (eDNA) in a biofilm treated with SCPN (without tobramycin and DNase I) (black line), Tob-SCPN (green line), and Tob-DNase I-SCPN (purple line). The biomass variations in (**a**, **b**) are represented as fold changes, while the variations in (**c**) are described as percentages. **d** Positions of nanoparticle aggregates in the biofilm matrix. The results presented in this figure are representative of an experiment repeated at least twice with similar results.

After 16 h of treatment, a 13 × 13 µm region of interest surrounding the nanoparticle aggregate was created to analyze the behavior in the biofilm components with time (see Methods). In that sense, Fig. 4 depicts the changes in matrix, cells and eDNA biomass in the area surrounding the nanoparticle aggregate. First, the innocuousness of the SCPNs was again demonstrated; bacteria grew exponentially and became stationary at 6–9 h when bacteria covered 100% of the region and could no longer spread (black line of Fig. 4a). Similarly, Fig. 4b shows the increase achieved during the time-lapse 16 h experiment by the extracellular matrix biomass in the control biofilm (treated with SCPN alone, black line). It is perhaps worth emphasizing that when a continuous biofilm was formed, the constant media flow through the chamber expelled the nonattached bacteria (Fig. 2b)^58^. However, during time-lapse imaging, the flow was stopped, and nonattached bacteria were allowed to grow planktonically in addition to the attached bacteria that continued to form the biofilm.

**Fig. 5:**
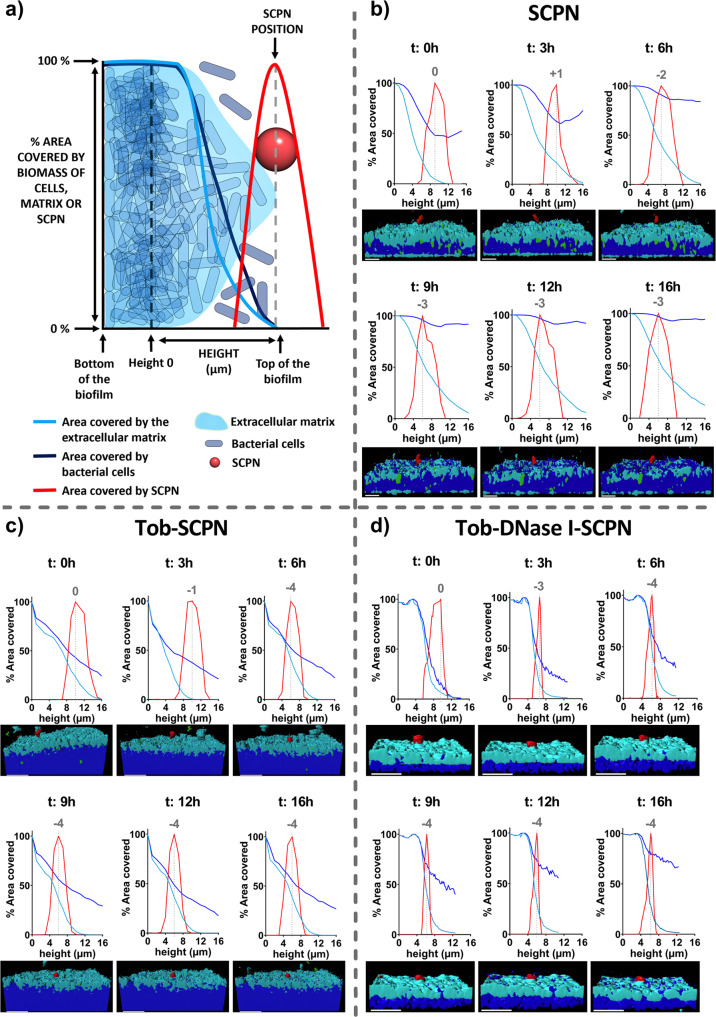
Tracking of an SCPN aggregate into the *P. aeruginosa* biofilm for 16 h. **a** Schematic representation of the graphs. **b** SCPN (without tobramycin and DNase I), (**c**) tobramycin-containing nanoparticles and (**d**) nanoparticles encapsulating both tobramycin and DNase I. Lines represent the area percentages occupied in layers by the cells (dark blue line), matrix (light blue line), and nanoparticles (red line) at different times. The height of 0 on the x-axis is taken as the moment when the biofilm started to decrease. The area values covered in the nanoparticle were normalized by assigning 100% at the highest percentage. Gray numbers represent movement (in µm) of the nanoparticle in the biofilm. A 3D representation view of the biofilm is illustrated for each time. The scale bar represents 10 µm. The results presented in this figure are representative microscopy images of an experiment repeated at least twice with similar results.

With all data recovered from the microscopic images of the time-lapse experiment, a 3D representation of the biofilm and a graphical representation for distribution of matrix biomass (extracellular matrix polysaccharides staining), cell biomass, eDNA, and nanoparticle aggregates in the biofilm were also performed (Fig. 5). To normalize the biofilms, all plots start at the x-axis in the upper part of the biofilm, when the area covered by cells and the extracellular matrix decreases. On the other hand, the area covered by nanoparticles was normalized by assigning 100% at the position where the most significant portion of SCPN aggregates were imaged. In the case of pristine SCPN (Fig. 5b), the bacterial cells grew in biofilm as well as planktonically, and the percentage of area occupied by bacterial cells (dark blue line) increased over time and included the whole area after 9 h. The 3D representation below the graphs depicts this planktonic growth with an increase in dark blue at the top of the biofilm (increase in bacterial cells). In the same figure, the progression of biofilm formation was also observed, as the area under the light blue curve was constantly growing during the experiment.

### DNase I prevents regrowth of the *P. aeruginosa* biofilm during 16 h of treatment

In the biofilm treated with SCPNs containing tobramycin alone or in combination with DNase I the biofilm cells and the extracellular matrix were sensitive and suffered apparent modifications. In both cases (Fig. 5c, d), biofilm matrix formation was impaired in the first hours, as the area under the light blue curve was lower than that for the control SCPN (Fig. 5b). Figure 4 also shows that biofilm treated with Tob-SCPN first decreased its total biomass, as shown by the green lines in Fig. 4a, b. However, increases in cell numbers and the extracellular matrix were observed after 6 and 12 h, respectively. Thus, enough tobramycin was available to fight bacteria at the start of the experiment (<6 h), but the concentration was not enough for the long term, and the living biofilm cells started to grow and build up the biofilm and the extracellular matrix again. These results might indicate that repeated doses (every 6–8 h) of Tob-SCPN will be required for efficient biofilm removal and treatment.

On the other hand, the biofilm treated with SCPNs containing both tobramycin and DNase I (Tob-DNase I-SCPN) underwent a progressive decrease in the extracellular matrix and a slight increase in the number of free bacterial cells (Fig. 4a, b, purple line). The tobramycin released from these SCPNs seems effective for the first 3 h of treatment, as the cell biomass started to decrease (Fig. 4a, purple line). Afterward, a significant increase in the bacterial cells was observed due to the delayed effect of DNase I, which started to disperse the biofilm by breaking down the internal eDNA network and releasing the bacterial cells. We observed that the tobramycin formulated with the nanoparticles actively killed free cells, as the slope of the purple line was much less pronounced than that corresponding to SCPN alone (Fig. 4a, black line). However, additional Tob-SCPN doses or higher concentrations of Tob-SCPN should be administered to avoid the spread of the biofilm^46^.

To observe equivalent SCPN aggregate sizes, similar amounts of the different SCPNs were administered to the biofilm for time-lapse imaging. However, it is important to point out that at the same nanoformulation weight, Tob-SCPN had a higher antibiotic concentration than Tob-DNase I-SCPN (Table 1). This higher tobramycin concentration indicated why biomass loss was faster with Tob-SCPN than with Tob-DNase I-SCPN (Fig. 4b, green line vs. purple line). Nevertheless, even with the higher concentration of tobramycin, regrowth of the biofilm was clearly observed after 12 h for the biofilm treated with Tob-SCPN. On the other hand, the biofilm treated with Tob-DNase I-SCPN continued to decrease over time until 16 h. Treatments with enzymes that degrade and breakdown the biofilm matrix, together with antibiotics that actively destroy or inhibit bacterial cells liberated from the biofilm, have been extensively studied, not only with deoxyribonucleases but also with proteases and glycoside hydrolases^59^. In summary, this combined treatment caused a reduction in the antibiotic dose necessary to treat the biofilm.

### Tob-DNase I SCPN enhances eDNA dispersion and interaction and penetrability into the *P. aeruginosa* biofilm matrix

As previously mentioned, SCPNs with sizes of ~20 nm are too small to be monitored with a confocal microscope (due to technical limitations), so the time-lapse experiment was carried out with SCPN aggregates with diameters of 1–2 µm. It must be pointed out that such aggregates are not representative of the individual behavior of SCPNs, but they allowed us to study their approach and interaction with the extracellular matrix. The Fig. 4d and the gray numbers in Fig. 5, illustrate the positions of nanoparticle aggregates in the matrix over time. In all cases, the nanoparticle aggregates were close to the biofilm surface, but in Tob-SCPN and Tob-DNase I-SCPN (green and purple lines of Fig. 4d and gray numbers in Fig. 5c, d), the nanoparticle aggregates moved 1 µm deeper inside the biofilm than SCPN alone. These nanoparticles, without any tobramycin or DNase I, were negatively charged (Table 1). Thus, electrostatic repulsions with the highly negatively charged extracellular matrix could be anticipated (black line in Fig. 4d, and gray numbers in Fig. 5b). These results are consistent with the penetration studies shown in Fig. 3, where penetration increased in the order SCPN < Tob-SCPN < Tob-DNase I-SCPN.

On the other hand, tobramycin exhibited considerably reduced biofilm diffusion because it is a positively charged antibiotic sequestered in the biofilm periphery by ionic interactions^60^. In some treatments, tobramycin is administered via an aerosol, as this mitigates toxicity, increases the final concentration at the infection site, and compensates for low diffusion into the biofilm^61^. However, this is not sufficient, and tobramycin and other antibiotics require enhanced biofilm diffusion and penetration to increase antimicrobial efficacy, especially in patients with CF and broadly chronic infections. Neutralization of the charges in tobramycin when formulated with negatively charged SCPNs, together with the DNase I released, could boost penetration inside the biofilms. Some authors have concluded that nanoparticle size is the key factor determining diffusion into the matrix^28^. Penetration of nanoparticles with sizes up to 70 nm has been observed, but the perfect size has been reported to be between 10 and 30 nm^62^. Since our SCPNs, formulated or not, have average sizes of ~20 nm (Table 1), they appear to be ideal candidates for penetration and diffusion inside the matrix.

Finally, the concentrations of eDNA surrounding the nanoparticle aggregates were also calculated over time. Clearly, eDNA was completely removed in <3 h for the biofilm treated with Tob-DNase I-SCPN (Fig. 4c, purple line), which demonstrated the efficacy of the enzyme. Interestingly, the biofilm treated with Tob-SCPN exhibited a constant decrease in eDNA levels, which was attributed to matrix disaggregation and release caused by tobramycin (Fig. 4c, green line). In contrast, the eDNA level gradually increased over time for the biofilm treated with SCPN (Fig. 4c, black line), which had already shown continuous growth and building of the extracellular matrix (Fig. 4b, black line). These results combined with the results obtained in Fig. 5, where Tob-SCPN and Tob-DNase I-SCPN showed deeper penetration, clearly demonstrated that biofilm matrix disaggregation improved the penetrability of the nanoparticles and enhanced antibiotic diffusion inside the biofilm, which will allow better treatment for chronic infections forming a biofilm, such as CF or COPD.

Many nanobased formulations have been developed to deliver antibiotics for chronic pulmonary infections and include inorganic nanoparticles, liposomes, polymeric nanoparticles, and hybrid systems, but most of these nanosystems have a low antibiotic encapsulation efficiency and were only tested in static biofilms, which do not resemble real infections in vivo. In this work, we loaded up to 40 wt% tobramycin in SCPNs based on natural dextran polysaccharides and functionalized them with the enzyme DNase I. These nanoformulations were tested against mature continuous-flow biofilms of *P. aeruginosa* and showed improved efficacy compared to treatment with free and soluble tobramycin and DNase I. Specifically, the use of SCPNs increased the biofilm reduction by 15% compared to the soluble treatment with the DNase I-SCPN. Moreover, with the Tob-DNase I-SCPN biofilm treatment, the biofilm biomass reduction achieved was 34 % using a very low quantity of antibiotic (2 µg/ml of tobramycin). Besides, we studied the detailed effects and interactions of the nanoformulations with the extracellular matrix components of *P. aeruginosa* biofilms with specific labeling of the different components and time-lapse imaging by laser scanning confocal microscopy. We demonstrated that the SCPNs neutralized the charges of tobramycin and, together with a DNase I formulation, boosted nanoantibiotic efficacy, penetrability and diffusion through the biofilm and avoided retention of the antibiotic at the biofilm periphery.

## Methods

### Materials

3-Mercaptopropionic acid (≥99%), 4-(4,6-dimethoxy-1,3,5-triazin-2-yl)-4-methylmorpholinium chloride (DMTMM·HCl) (96%) and rhodamine B (97%) were purchased from Aldrich. Phosphate-buffered saline (PBS) was purchased from Scharlau. 4-(Dimethylamino)pyridine (DMAP) and tobramycin (97%) were purchased from Acros-Organics. DNase I was purchased from Panreac, Applichem. The water (H_2_O) used in the syntheses was deionized water from a Milli-Q A10 Gradient system (Millipore).

### SCPN: dextran-based single-chain polymer nanoparticles labeled with rhodamine and functionalized with 3-mercaptopropionic acid

DXT-SCPN (100 mg, 484 µmol glucose) was dispersed into pH 7.4 PBS at 10 mg/mL for 1 h. Then, a mixture of DMTMM·HCl (4.6 mg, 16.7 μmol) and rhodamine B (5.3 mg, 11 μmol) were added to PBS, and the reaction was stirred overnight at room temperature. The reaction was monitored by TLC in methanol to ensure correct functionalization of the particles by rhodamine. The methacrylate groups of the particles were functionalized by reaction with 3-mercaptopropionic acid (117 mg, 1.11 mmol) at pH 9 for 5 h. The crude material was purified by dialysis (3.5 kDa MWCO RC membranes) against water. Water was refreshed twice a day until the water conductivity reached a value below 1 μS/cm. The final product was freeze-dried and kept at 4 °C until further analysis. Yield: 93%. The glucose substitution degree DS = 45%. The cross-linking degree CL = 5%. Mw = 59 kDa. The Rhodamine B content was 18 μg/mg nanocarrier. Dh (DLS) = 19 ± 1 nm; PdI = 0.26 ± 0.04; zeta potential (pH = 7, 25 °C) = −21 ± 1 mV; Dn (TEM, uranyl staining) = 21 ± 8 nm.

^1^H NMR (500 MHz, D_2_O) δ ppm: 8.55–6.74 (m, 0.3 H, rhodamine H_ar_), 5.54–4.86 (2.6 H, including H-1 and H-2/3 substituted Glc), 4.25–3.33 (11.6 H, m, rest of Glc and CH_2_ Rh), 1.28 (s, 3H, CH_3_ Gluc, and CH_3_ Rh) (Supplementary Fig. 4).

### DNase I-SCPN

SCPN (10 mg, 0.2 μmol) and DNase I (11.3 mg, 0.4 μmol) were dispersed into water at 10 mg/mL at pH 7 and mixed for 1 h at room temperature. The sample was freeze-dried before further analysis. Dh (DLS) = 102 ± 2 nm; PdI = 0.52 ± 0.01; zeta potential (pH = 7, 25 °C) = −28 ± 1 mV; Dn (TEM, uranyl staining) = 20 ± 5 nm.

### Tob-SCPN

SCPN (6 mg, 0.1 μmol) and tobramycin (4 mg, 8.6 μmol) were dispersed and dissolved into water at 10 mg/mL separately, and the pH was adjusted to 7. Then, both were mixed and stirred for 1 h at room temperature. The sample was freeze-dried and kept at 4 °C until further analysis. Dh (DLS) = 17 ± 1 nm; PdI = 0.27 ± 0.01; zeta potential (pH = 7, 25 °C) = −5 ± 2 mV; Dn (TEM, uranyl staining) = 37 ± 13 nm.

### Tob-DNase I-SCPN

Tob-SCPN (12.4 mg) and DNase I (11.2 mg) were dispersed into 1 mM NaCl at 10 mg/ml at pH 7 and gently mixed for 1 h at RT. Dh (DLS) = 11 ± 1 nm; PdI = 0.27 ± 0.07; zeta potential (pH = 7, 25 °C) = −6 ± 1 mV; Dn (TEM, uranyl staining) = 37 ± 13 nm.

### Dynamic light scattering (DLS)

DLS analyses were conducted using a Zetasizer Nano ZS, ZEN3600 Model (Malvern Instruments Ltd). All measurements were performed in disposable sizing cuvettes at a laser wavelength of 633 nm and a scattering angle of 173°, while the zeta-potential measurements were performed in disposable zeta-potential cells (pH 7.4, 25 °C). Before the measurements, samples were dispersed at concentrations of 10 mg/mL in PBS solution for size measurements and 1 mM NaCl for zeta-potential measurements. Each sample measurement was repeated three times at 25 °C.

### Nuclear magnetic resonance (^1^H NMR)

NMR spectra were recorded on a Bruker AVANCE III spectrometer at 500 MHz and 25 °C. Chemical shifts (δ) are given in ppm relative to the residual signal of the solvent. Splitting patterns: b, broad; s, singlet; d, doublet; t, triplet; q, quartet; m, multiplet.

### UV–vis

UV–vis measurements used to determine rhodamine loading were carried out at 1 mg/mL in PBS using a UV-2401PC UV–vis recording spectrophotometer from Shimadzu. The rhodamine B content was calculated using a calibration curve for rhodamine B in PBS.

### Liquid chromatography–mass spectrometry (LC–MS)

LC–MS data were recorded using a UPLC Acquity instrument equipped with an Acquity C18 100*2.1 mm*1.7 μm column coupled to an LCT Premier XT ESI-TOF mass spectrometer (source: electrospray positive mode with a mode m/z range 50-1000, capillary vol: 1000/cone volt: 50) and analyzed using Masslynx 4.1 software from Waters. The mobile phases used were A: 0.1% HFBA in water and B: 0.1% HFBA in acetonitrile. Tobramycin elution was carried out isocratically with 95% A for 2 min and a gradient from 95 to 1% An over 15 min. Tobramycin was detected after 6.7 min elution (*m*/*z* = 468).

### Transmission electron microscopy (TEM)

TEM analyses were performed in a TECNAI G2 20 TWIN microscope (FEI, Eindhoven, The Netherlands) operating at an accelerating voltage of 200 KeV in bright-field image mode. One drop of the sample dispersion in water (~3 μL, 0.1 mg/mL) was deposited on a carbon film supported on a copper grid (300 mesh) and hydrophilized by a glow discharge process just before use. After staining for 20 s with a uranyl acetate aqueous solution (1% w/v), the sample was dried at room temperature overnight. The number-average diameter was calculated by ImageJ platform analysis using Gaussian curve fitting after counting at least 100 nanoparticles.

### Bacterial strains and growth conditions

Wild-type *Pseudomonas aeruginosa* PAO1 CECT 4122 (ATCC 15692) was obtained from the Spanish Type Culture Collection (CECT) and cultivated at 37 °C in Luria-Bertani broth medium (LB) (Scharlab). Bacterial growth was measured by measuring the optical density (O.D.) at 550 nm.

### Antibacterial activity against planktonic bacteria

*P. aeruginosa* PAO1 was grown planktonically to the initial exponential log phase (O.D._550 nm_ ≈ 0.1) and plated in a microtiter plate (Corning 3596 Polystyrene Flat Bottom 96 Well) supplemented with different SCPN and antibiotic concentrations following the Clinical Laboratory Standards Institute (CLSI) guidelines^63^. As previously described^45^, the microtiter plate was incubated at 37 °C in a SPARK Multimode microplate reader (Tecan) with 150 rpm shaking. Bacterial growth was monitored for 8 h by reading the OD_550 nm_ every 15 min.

### DNase I activity testing

DNase I-SCPN and a known DNase I (Panreac Applichem, ref A3778) concentration were incubated with 100 ng of *P. aeruginosa* genomic DNA for 30 min at 37 °C. Samples were then loaded into a 0.8% agarose gel plus ethidium bromide (0.25 µg/mL) and visualized under U.V. light in a Gel Doc^TM^ XR+ (Bio–Rad Laboratories). DNase I activity was estimated with DNA degradation quantification performed with Quantity One software (Bio–Rad Laboratories).

### Antibacterial activity against continuous-flow biofilms

Continuous *P. aeruginosa* biofilms were grown using a flow-cell system, as previously reported^45,64^. Biofilms were grown with LB medium plus 0.2% glucose to stimulate biofilm formation, and the flow cells were inoculated with 350 µl of *P. aeruginosa* PAO1 at an OD_550 nm_ ≈ 0.1. After allowing the bacteria to attach for 2 h, media was pumped through the system at 42 µL/min employing an Ismatec ISM 943 peristaltic pump (Ismatec). Bubble traps (DTU Systems Biology) were placed in the system to avoid biofilm disturbances. Biofilms were grown for 3 days (72 h) at room temperature, and then the treatment (0.26 µg/mL DNase I, 2 µg/mL tobramycin or the equivalent nanoformulation concentration (5 µg Tob-SCPN/mL and 8.7 µg Tob-DNase I-SCPN/mL)) was injected into the mature biofilms. After 16 h of treatment, biofilms were stained with SYTO9 and propidium iodide (Live/Dead BacLight Bacterial Viability Kit, Thermo Fisher Scientific) following the manufacturer’s instructions and visualized using a Zeiss LSM 800 confocal laser scanning microscope (CLSM) with a 20×/0.8 air objective. Biomass and thickness quantification of the images obtained was performed using FIJI and COMSTAT2 software^65^.

### Franz cell diffusion assay

The diffusion assay was carried out by using 5 mL Franz cells (PermeGear). The receptor and donor chambers were divided by means of a polyethersulfone (PES) hydrophilic membrane with a pore size of 0.22 µm and a 13 mm diameter (Millipore Express^®^ PLUS Membrane Filters, Merck) that was applied between the two compartments. Before applying the membrane between the cell compartments, the *P. aeruginosa* biofilm was grown at 37 °C for 72 h on the membranes. The receptor chamber was filled with 5 mL PBS. A suspension of the controls and formulations to be tested (Tob, SCPN, Tob-SCPN and Tob-DNase I-SCPN) were uniformly added to the top of the biofilm. The cell system was incubated at room temperature under continuous stirring, and samples (1 mL) were removed at defined times (1, 2, and 4 h). The tested SCPNs were labeled with rhodamine as previously described. Samples were analyzed for tobramycin content by LC–MS and SCPN content by fluorescence analysis using a Synergy HT microplate reader (Biotek Instruments). The results are reported as the percentage of SCPNs that diffused through the whole biofilm layer and were quantified in the receptor chamber of the Franz Cell.

### Time-lapse experiments

Continuous *P. aeruginosa* PAO1 biofilms were grown in flow-cell chambers as previously explained. To obtain time-lapse images, 100 µg/mL nanoparticles were injected into mature-grown *P. aeruginosa* PAO1 biofilms (72 h old) with specific biofilm staining: DAPI at 300 nM (Life Technologies) for intracellular DNA (biomass), TOTO™-1 iodide at 1 µM (Thermo Fisher) to stain eDNA, and concanavalin A AlexaFluor™ 647 conjugate at 100 µg/mL (Life Technologies) to dye the exopolysaccharides of the biofilm extracellular matrix. Microscopic images were taken approximately every 3 h with a 63×/1.4 oil objective mounted in a Zeiss LSM 800 CLSM using the multiposition Time Series module of ZEN software and the Definite Focus.2 system implemented in our confocal microscope (Zeiss). Analysis of matrix, cells, eDNA, and nanoparticle variation was made by creating a specific ROI (region of interest) of 13 × 13 µm around the nanoparticle and quantifying the biomass and area occupied by layers using FIJI and COMSTAT2 software. 3D representation was created using IMARIS software (Bitplane AG).

## Supplementary information

Supplemental Material

## Data Availability

The authors declare that all relevant data supporting the findings of the study are available within the article and the Supplementary Information file. Additional details can be available from the corresponding author upon reasonable request.
