# Supplementary material for: Neutralization of ionic interactions by dextran-based single-chain nanoparticles improves tobramycin diffusion into a mature biofilm
Source: NPJ Biofilms Microbiomes. 2022 Jul 4;8:52. doi: 10.1038/s41522-022-00317-9 (PMC9253323; doi:10.1038/s41522-022-00317-9)
Supplement: Supplementary file 1 — Supplemental Material [file 41522_2022_317_MOESM1_ESM.pdf]

SUPPLEMENTARY INFORMATION

Neutralization of ionic interactions by dextran-based single-chain nanoparticles improves tobramycin diffusion into a mature biofilm

Núria Blanco-Cabra[1,2,*], Julie Movellan[3], Marco Marradi[3,4], Raquel Gracia[3], Cristian Salvador[3], Damien Dupin[3], Iraida Loinaz[3], Eduard Torrents[1,2,*]

Physico-chemical properties of pharmagrade dextran 40 kDa used as precursor for the synthesis of DXT-SCPNs

Dh (DLS, 10 mg/mL in PBS) = 11 ± 1 nm, PdI = 0.19 ± 0.05

Data from batch release certificate: Mw = 42208 Da, Mw/Mn = 1.3

Supplementary Figure 1. Functionalization of DXT-SCPN with Rhodamine and 3-mercaptopropionic acid (MPA). DXT is a branched polymer but is drawn as linear for reasons of clarity.

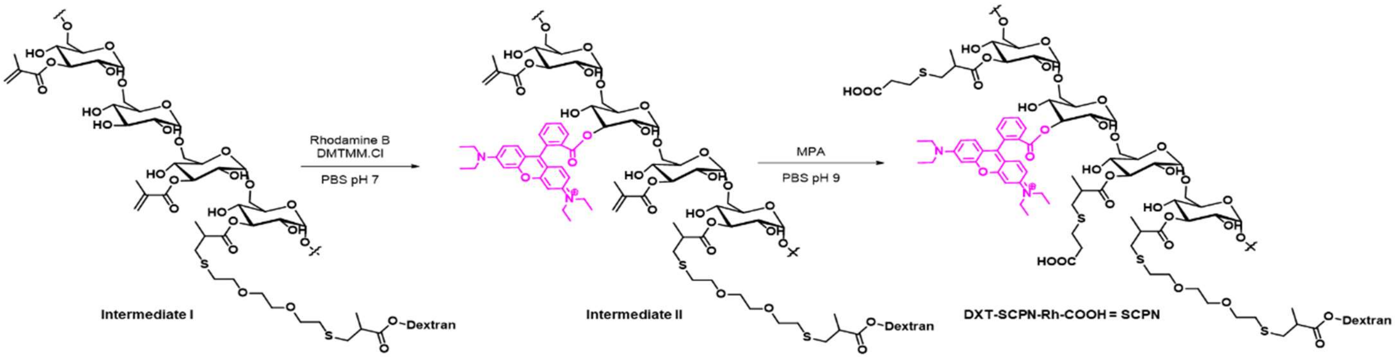

Supplementary Figure 2. ^{1}H NMR spectrum of rhodamine-labelled SCPN in D_2O

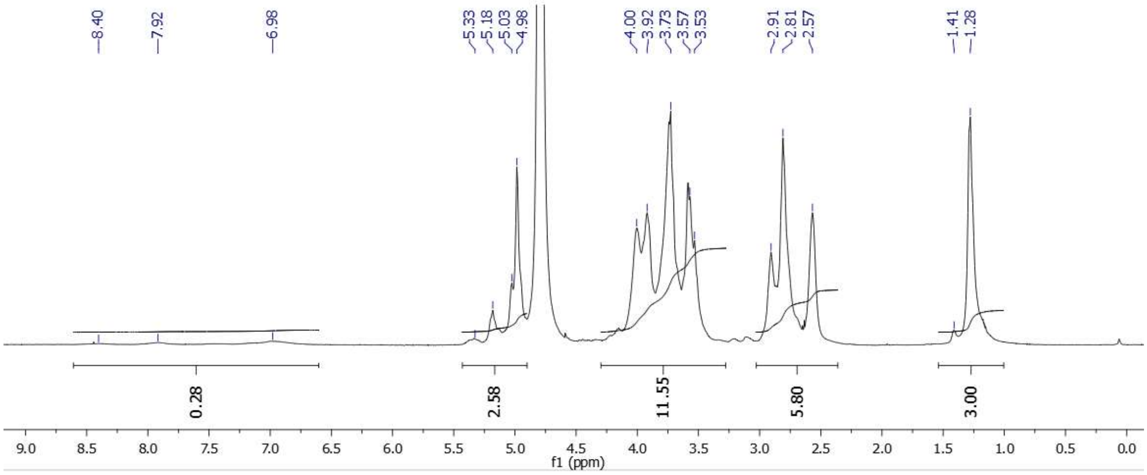

Supplementary Figure 3. UV–vis calibration curve for Rhodamine B in PBS.

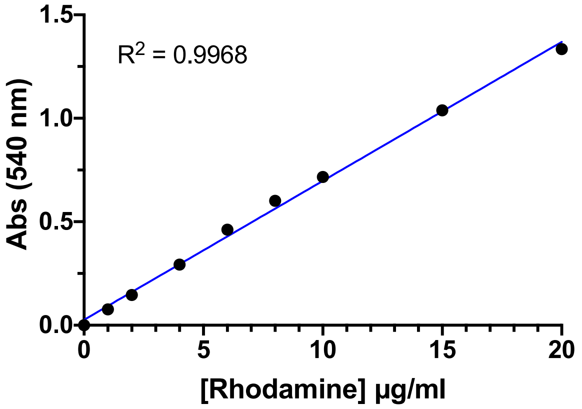

Supplementary Figure 4. ^{1}H NMR spectra of SCPN, DNase I-SCPN, Tob-SCPN, Tob-DNase I-SCPN, DNase I, and Tobramycin in D_2O.

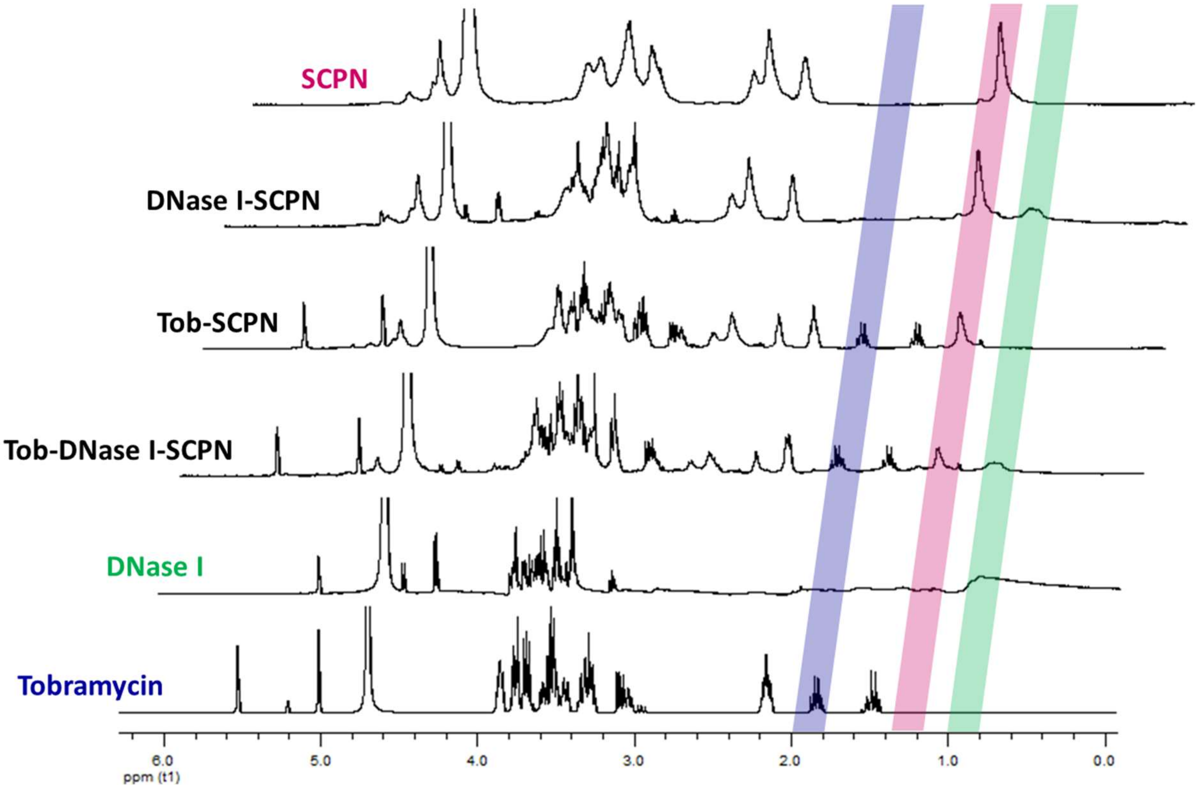

Supplementary Table 1. Percentage of dead bacteria biomass (stained in red in the Live/Dead BacLight Bacterial Viability Kit) in the total biofilm biomass of the 72-hour-old biofilms of *P. aeruginosa* treated for 16 hours with 0.26 µg/ml DNase I and/or 2 µg/ml tobramycin and with nanoformulation containing SCPN with the corresponding concentration of both actives. Refer to figure 2 for microscopic images.

	**CONTROL**	**SCPN**	**Tob + DNase I**	**Tob + DNase I SCPN**	**Tob**	**Tob SCPN**	**DNase I**	**DNase I SCPN**
**% Dead biomass / Total biomass**	5,57 % ± 0,86	5,67 % ± 0,32	9,75 % ± 0,64	12,44 % ± 0,13	9,56 % ± 0,47	12,29 % ± 0,61	7,14 % ± 0,52	8,54 % ± 0,26
